# Utility of 99mTc-Sestamibi Single-Photon Emission Computed Tomography (SPECT)/CT Single Imaging Strategy in the Preoperative Localization of Parathyroid Adenoma

**DOI:** 10.7759/cureus.51828

**Published:** 2024-01-07

**Authors:** Nikhil Vasandani

**Affiliations:** 1 Surgery, Royal College of Surgeons In Ireland, Dublin, IRL

**Keywords:** 4d ct, 99mtc sestamibi scintigraphy, minimally invasive parathyroidectomy, parathyroid gland adenoma, mri images, single photon emission tomography (spect), ultrasound (u/s), parathyroid cancer

## Abstract

Background

Primary hyperparathyroidism is an endocrinopathy associated with dysregulated calcium homeostasis. The most common etiology is a parathyroid adenoma most definitely managed via a parathyroidectomy. The two main surgical approaches include a minimally invasive parathyroidectomy (MIP) and open four-gland exploration (4-GE). MIP is the preferred operative strategy since it is associated with less postoperative complications. Accurate preoperative imaging is essential in informing the optimal approach to surgery. MIP is only considered if adenoma is able to be localized precisely. The most commonly used imaging modality includes ultrasound and sestamibi single-photon emission computed tomography (SPECT)/CT, either as a single or combination strategy. Other options include MRI, PET, and 4D CT. There is no universally accepted preoperative imaging strategy. The literature is discordant and recommendations proposed by existing guidelines are incongruous.

Objectives

This study aimed to evaluate currently utilized preoperative parathyroid imaging modalities at our institution and correlate them with surgical and histological findings to determine the most efficient imaging strategy to detect adenomas for our patient cohort. This will ultimately guide the best surgical approach for patients receiving parathyroidectomies.

Methods

This is a retrospective observational study of all patients undergoing first-time surgery for biochemically proven primary hyperparathyroidism at our institution over the past five years. Multiple data points were collected including modality of preoperative disease localization, operation type, final histopathology, biochemical investigations, and cure rate. Patients were categorized into one of three groups based on the method of disease localization.

Results

A total of 244 patients had parathyroidectomies performed at our institution in the past five years from January 2018 to December 2022. Ninety-six percent (n=235) of all patients received dual imaging preoperatively with SPECT/CT and ultrasound performed on the same day and therefore included in this study. A total of 64.3% (n=151) underwent MIP. Eighty percent (n=188) of all histopathology revealed adenomas and 26.8% (n=63) of patients had adenoma localized on SPECT/CT only (sensitivity: 58.1%, specificity: 71%, and positive predictive value {PPV}: 85.7%). A total of 9.8% (n=23) had adenoma localized on ultrasound only (sensitivity: 15.6%, specificity: 73.3%, and PPV: 65.2%). A total of 45.1% (n=106) were dual localized on both SPECT/CT and ultrasound (sensitivity: 75.6%, specificity: 46.6%, and PPV: 84.9%). The cure rate was 91.5% in the dual-localized group, 86% in the dual-unlocalized group, and 96.5% when localized with SPECT/CT alone.

Conclusion

A dual-imaging modality with SPECT/CT and ultrasound should remain the first-line imaging strategy. This approach has higher sensitivity rates and poses no inherent patient or surgical-related risks. Patients with disease unlocalized on SPECT/CT alone had a positive predictive value, specificity, and likelihood ratio for adenoma detection comparable to dual-localized patients. Therefore, SPECT/CT alone is sufficient for directing MIP in the presence of a negative ultrasound.

## Introduction

Primary hyperparathyroidism (PHPT) is an endocrinopathy associated with the autonomous hypersecretion of parathyroid hormone (PTH) from one or more parathyroid glands, resulting in dysregulated calcium homeostasis [1].

It has an estimated incidence of 21-57 cases per 100,000 individuals per year and is most commonly diagnosed in adulthood [2]*.* Globally, PHPT exhibits a prevalence of 0.84%, with a predisposition towards females and individuals of African-American descent [3]. Eighty percent of affected individuals are asymptomatic while the remainder present with features of hypercalcemia [4]*.* PHPT is most commonly associated with PTH-producing adenomas (PTA). This can be sporadic or caused secondary to familial pathologies like MEN1 syndrome [5]*.*

Early diagnosis and appropriate management of PHPT are vital to prevent long-term consequences of hypercalcemia. The most definitive strategy for managing adenomas includes performing a parathyroidectomy. This can be performed minimally invasively (MIP) or via an open, four-gland exploratory approach (4-GE) [6]*.*

MIP offers several advantages, including reduced surgical duration, superior scar cosmesis, shorter postoperative hospitalization, improved pain management, and diminished incidence of postoperative hypocalcemia. Consequently, MIP has emerged as the favored surgical method among clinicians [7].

Accurate preoperative localization of adenomatous glands is an indispensable prerequisite for justifying the adoption of a minimally invasive approach, as the initial operation plays a pivotal role in achieving a definitive cure and total treatment response [8]. Gold standard treatment for adenoma-driven PHPT includes accurate preoperative disease localization and performance of a successful, totally curative MIP at first operation [9]*.* Numerous imaging modalities are available for localizing parathyroid pathology. Common options include ultrasound and 99mTc-sestamibi scintigraphy combined with various disease capture techniques, such as computed tomography (CT), four-dimensional CT (4D CT), magnetic resonance imaging (MRI), positron emission tomography (PET), and a variety of radiotracers [10]*.* Of these, ultrasound and 99mTc-sestamibi single-photon emission computed tomography (SPECT)/CT are the predominant preoperative parathyroid imaging modalities in clinical practice [11]*.*

A comprehensive review of the existing literature on this subject matter reveals a notable discrepancy in the recommendations for optimal preoperative disease localization strategies. Divergent research findings have led to a lack of consensus. Presently, the primary guidelines governing preoperative imaging practices are those formulated by the National Institute for Health and Care Excellence (NICE) and the European Association of Nuclear Medicine (EANM) [12,13].

NICE guidelines advocate for ultrasound as the initial localization modality, with a subsequent sestamibi scan recommended if localization fails [12]. Conversely, EANM guidelines propose a combined approach involving single-isotope dual-phase 99mTC-sestamibi SPECT/CT and ultrasound [13].

Currently, at our institution, almost all patients receive dual imaging with both single-isotope dual-phase 99mTC-sestamibi SPECT/CT and ultrasound performed on the same day in concordance with EANM guidelines [13]. The decision to proceed with MIP is contingent upon dual localization on both imaging modalities.

Emerging findings in the current literature challenge the effectiveness of ultrasound as a routine preoperative requirement for localizing disease [14]. Consequently, the primary objective of this study was to determine if a single-imaging strategy with SPECT/CT alone is as efficacious in localizing disease relative to a dual-imaging strategy combining SPECT/CT and ultrasound. To improve the readability of the article, whenever a SPECT/CT is mentioned, it is implied that a 99mTC-sestamibi SPECT/CT is in question.

## Materials and methods

A retrospective observational study was performed on all the parathyroidectomies performed at our institution over the past five years from January 2018 to December 2022. Local institutional ethical approval was not required as this is a retrospective observational study. All sensitive patient information was excluded and all data was anonymized as per institutional standards and protocols.

Our participant inclusion criteria included patients undergoing first-time surgery for biochemically proven PHPT who received both US and 99mTC-sestamibi SPECT/CT preoperatively as a mode of disease localization. Patients managed conservatively, those with a history of prior thyroid or parathyroid surgery, and those afflicted with secondary and/or tertiary hyperparathyroidism were excluded from the analysis (Table 1).

**Table 1:** Inclusion and exclusion criteria of the present study. PHPT: primary hyperparathyroidism; US: ultrasound; SPECT: single-photon emission computed tomography

Inclusion criteria	Exclusion criteria
Biochemically proven PHPT	Secondary or tertiary hyperparathyroidism
First-time surgery	Prior thyroid/parathyroid surgery
Received both US and SPECT/CT preoperatively	Conservatively managed patients

An extensive parathyroidectomy database was constructed on Microsoft Excel 2016 which included a collection of multiple data points of all qualifying patients. This included demographic data like age, preoperative biochemical investigations like PTH, adjusted calcium, estimated glomerular filtration rate (eGFR), vitamin D, and urinary calcium. Modality of preoperative disease localization was recorded as patients who had disease localized on US only, SPECT/CT only, or on both ultrasound and SPECT/CT. The surgical approach of choice (MIP or 4-GE) of all patients was recorded. The histopathological findings following surgery were documented and categorized as parathyroid adenoma, hyperplasia, indeterminate, or normal. Postoperative PTH and adjusted calcium were also documented for each patient to establish treatment response and cure rate.

All demographic data was collected manually using the hospital's electronic medical record system. Information regarding preoperative and postoperative biochemical investigations was obtained using the local laboratory system. All histopathology of qualifying patients was obtained from the pathologist. All ultrasound and SPECT/CT findings were obtained using reports commented on by consultant radiologists. A double data entry system was put in place to ensure the integrity of the captured data.

**Figure 1:**
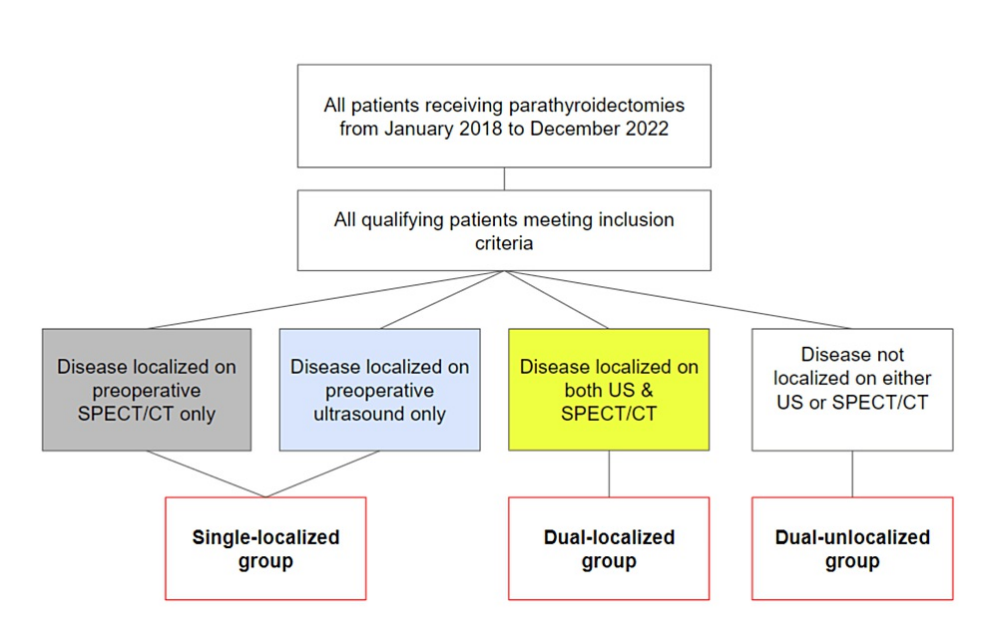
Flowchart outlining patient cohorts based on the method of disease localization. SPECT: single-photon emission computed tomography; US: ultrasound

Following data collection, all patients were stratified into one of three distinct groups predicated on the modality of disease localization. Patients localized on both SPECT/CT and ultrasound were put in the "dual-localized cohort," whereas those localized using either SPECT/CT or ultrasound exclusively were allocated to the "single-localized cohort." Those with disease localized on neither SPECT/CT nor ultrasound were placed in the "dual-unlocalized group" (Figure 1).

A statistical analysis was performed using Jamovi version 2.3.26.0. Comprehensive descriptive statistics, encompassing sensitivity, specificity, power calculations, predictive values, and likelihood ratios were performed. All figures were generated using Google Sheets.

It is important to note that since this was a fully anonymized retrospective observational study, ethical considerations did not necessitate seeking informed consent from patients before the commencement of the project. However, all patients generally consented to the potential use of their data for further research as part of the standard preoperative consent process for all procedures at our institution.

## Results

A total of 244 patients had parathyroidectomies performed at our institution in the past five years from January 2018 to December 2022. Among these patients, a noteworthy 96% (n=235) received preoperative dual imaging with single-isotope dual-phase 99mTC-sestamibi SPECT/CT and US performed on the same day. The remaining 4% (n=9) had data missing or received single modality imaging with SPECT/CT or US alone. Subsequently, 235 patients fulfilled the study inclusion criteria.

**Figure 2:**
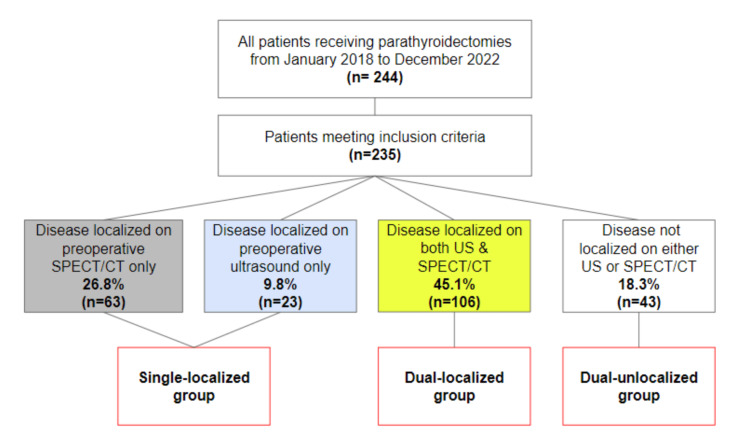
Flowchart depicting adenoma incidence based on the method of disease localization. SPECT/CT: single-photon emission computed tomography; US: ultrasound

Out of the 235 included patients, 63 patients (26.8%) had disease localized on SPECT/CT only and 23 patients (9.8%) had disease localized only on ultrasound. The majority of patients (45.1%) had disease localized on both SPECT/CT and ultrasound. Forty-three patients (18.3%) were not localized on either ultrasound or SPECT/CT (Figure 2).

The collective cohort exhibited an average reported age of 51 years, with the female gender constituting the majority at 71% (n=167). A total of 64.3% (n=151) of patients received MIP while the remainder 35.7% (n=84) received 4-GE. Postoperative histopathological analysis revealed adenomas in 80% (n=188) of all parathyroidectomy cases, hyperplasia in 5.1% (n=12), normal findings in 9.8% (n=23), and indeterminate pathology in 5.1% (n=12) of cases. A subset analysis looking at sensitivity, specificity, predictive values, and likelihood ratios for adenoma detection was performed relative to the modality of disease localization (Table 2).

**Table 2:** Subset analysis of adenoma detection based on method of disease localization. US: ultrasound; SPECT/CT: single-photon emission computed tomography

Statistic	Single-localized (US only)	Single-localized (SPECT/CT only)	Dual-localized (SPECT/CT + US)
Positive predictive value (PPV) for adenoma localization	65.2%	85.7%	84.9%
Sensitivity for adenoma localization	15.6%	58.1%	75.6%
Specificity for adenoma localization	73.3%	71.0%	46.6%
Likelihood ratio for a positive test result	0.58	2.00	1.40
Likelihood ratio for a negative test result	1.15	0.59	0.52

## Discussion

**Figure 3:**
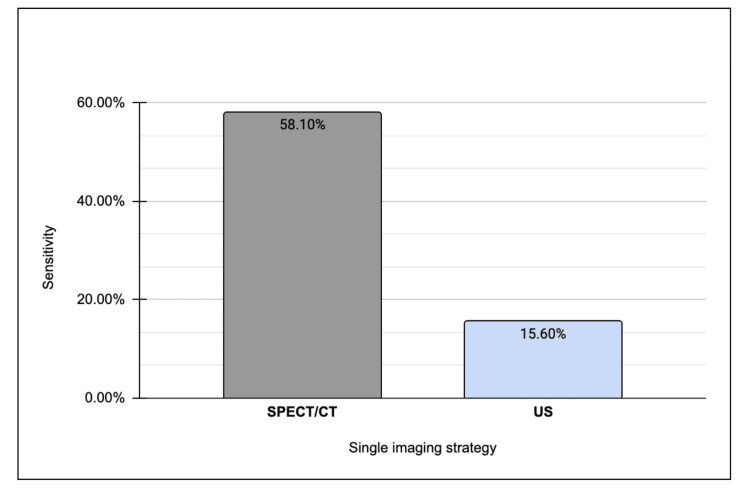
Sensitivity of adenoma localization on single SPECT/CT versus ultrasound. SPECT/CT: single-photon emission computed tomography; US: ultrasound

**Figure 4:**
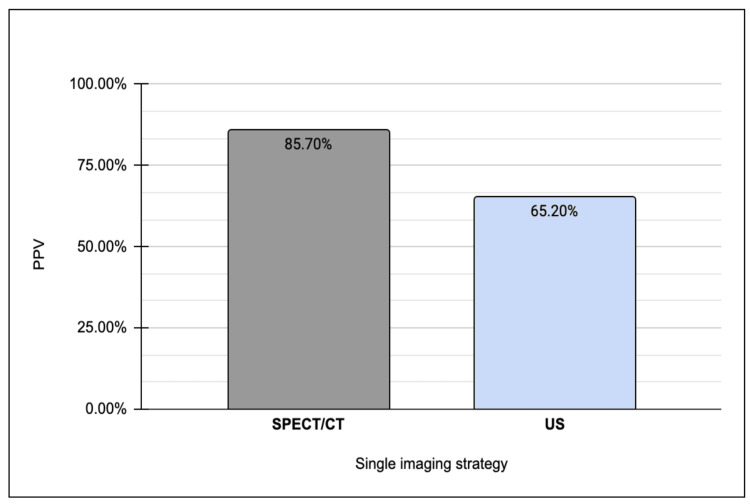
Positive predictive value of adenoma localization on single SPECT/CT versus ultrasound. PPV: positive predictive value; SPECT: single-photon emission computed tomography; US: ultrasound

After processing all the data, a comparative analysis between all three patient cohorts was conducted. When directly comparing detection rates between those with disease localized on SPECT/CT only relative to US, it was determined that localization using SPECT/CT demonstrates a nearly fourfold increase in sensitivity compared to sole reliance on ultrasonography, while maintaining a commensurate level of specificity for adenoma detection (Figure 3). Moreover, SPECT/CT exhibits a heightened positive predictive value in adenoma localization relative to US (Figure 4).

It is noteworthy that the likelihood of identifying an adenoma in the presence of a positive SPECT/CT result is nearly four times more probable in comparison to US alone. Consequently, if a singular imaging strategy were to be employed, SPECT/CT is recommended as the preferred first-line imaging modality over US. This recommendation engenders considerable debate, as it contravenes the prevailing guidelines put forth by the National Institute for Health and Care Excellence, which advocates for US as the primary imaging modality of choice [12].

**Figure 5:**
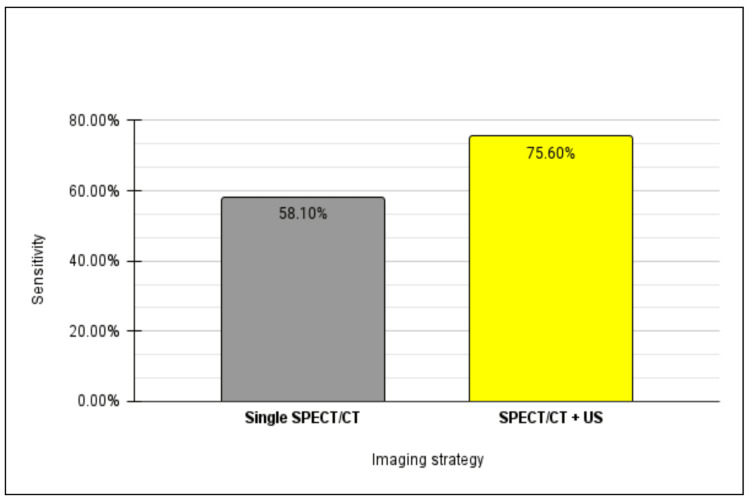
Sensitivity of adenoma localization on single SPECT/CT versus dual imaging. SPECT: single-photon emission computed tomography; US: ultrasound

**Figure 6:**
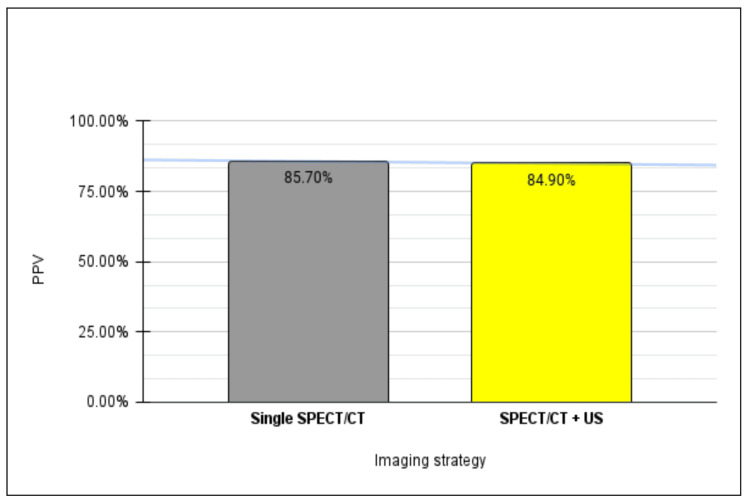
Positive predictive value of adenoma localization on single SPECT/CT versus dual imaging. PPV: positive predictive value; SPECT: single-photon emission computed tomography; US: ultrasound

Furthermore, upon comparing patients whose disease was localized on both SPECT/CT and US with those solely on SPECT/CT, our analysis revealed that dual-image localization yielded 1.3 times higher sensitivity for adenoma detection compared to SPECT/CT alone (Figure 5). In contrast, a converse observation was made regarding adenoma specificity between the two strategies, with a single-localizing SPECT/CT exhibiting 1.5 times greater specificity relative to its dual-localizing counterpart. Moreover, the PPV for adenoma detection was found to be comparable between single-imaging SPECT/CT and dual-imaging, standing at 85.7% and 84.9%, respectively (Figure 6).

Our analysis revealed that the probability of identifying an adenoma in the presence of a positive SPECT/CT result alone is nearly 1.5 times higher than disease localized on dual imaging. Furthermore, the postparathyroidectomy cure rate was observed to be 5% higher in the single SPECT/CT cohort compared to the dual-localized group.

It can be devised from this descriptive analysis that SPECT/CT alone, despite offering similar sensitivity, exhibits superior specificity, positive predictive value, likelihood ratio, and cure rate when contrasted with patients with disease localized on both modalities. These findings lead to the conclusion that a single-localizing SPECT/CT remains equally effective for adenoma localization relative to a dual-imaging strategy. Consequently, the consideration of MIP can be retained among patients with a positive SPECT/CT result in the presence of a negative US. This debates the recommendations outlined in the EANM guidelines, which advocate for second-line imaging strategies in the event of non-dual localization before MIP, as well as NICE guidelines, which propose a 4-GE approach in cases of discordant results between both imaging [12,13].

In the subset of patients where the disease was localized using both SPECT/CT and US, a substantial 86.8% (n=92) of individuals underwent MIP, while the remaining 13.2% (n=14) received 4-GE. This 13.2% is presumed to represent a converted patient cohort, initially scheduled for MIP but subsequently shifted to 4-GE, either due to intraoperative failure of disease localization or the emergence of other intraoperative complications necessitating an exploration.

**Figure 7:**
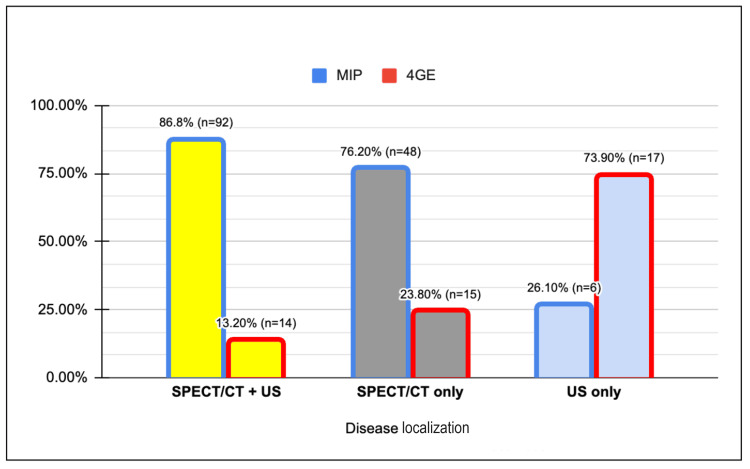
Surgical approach based on modality of preoperative disease localization. MIP: minimally invasive parathyroidectomy; 4-GE: four gland exploration; SPECT: single-photon emission computed tomography; US: ultrasound

For patients with disease localized exclusively via SPECT/CT, coupled with a negative result in US, a notable 76.2% (n=48) received MIP. It is noteworthy that there was a 10% lower rate of MIP performed in this group relative to those who were dual localized. Interestingly, this same group demonstrated a 10% higher incidence of 4-GE (Figure 7).

In light of our data, it is apparent that by converting a greater number of patients with single localizing SPECT/CT to potential MIP candidates, we have the power to increase the rate of MIPs performed by an average of 10%.

It may be argued that if our data-driven analysis suggests that dual imaging yields only marginal additional benefits compared to a single-localizing SPECT/CT, then what justifies its retention as the primary imaging modality, instead of transitioning to a singular imaging approach with SPECT/CT alone?

A single-parathyroid ultrasound scan costs our institution 160 USD and takes approximately 30 minutes to an hour to perform [15,16]. Some might consider performing an additional preoperative US without demonstrable added value as a misallocation of hospital resources and fiscal expenditure, introducing substantial inefficiency by impeding timely healthcare delivery. However, it is imperative to acknowledge that the dual-imaging cohort displayed a 17% higher sensitivity rate for adenoma detection relative to SPECT/CT single-localized patients. US results are subject to operator-dependent variability, with outcomes occasionally contingent on the proficiency and expertise of the sonographer which can be further improved [17]. In addition, US is a non-invasive modality, and its integration as a standard in preoperative parathyroid imaging carries no inherent risk to the patient, ultimately justifying its continued use in clinical practice [18]*.*

Limitations

The following study employs a retrospective observational design. This employs inherent biases and limitations associated with the analysis of historical data. Prospective studies with predefined protocols could offer more robust evidence. This could be a potential target for a future study. Moreover, all the study data was exclusively derived from a single institution which might not be representative of broader populations or practices. The findings of this study were not validated against external datasets which poses a selection bias that could restrict the generalizability of the findings and the applicability of the recommendations. Performing a multicenter study could help improve this limitation and eliminate bias.

As alluded to previously, the efficacy of ultrasound in adenoma localization is intrinsically subjective and markedly contingent upon the skill and technique of the sonographer [17]. This could have influenced the accuracy of disease localization leading to higher or lower detection rates based on the performing sonographer. This is a bias that directly influences ultrasound’s sensitivity and specificity rates. Repeating the study in a more standardized manner ensuring that all ultrasounds are performed by a single-identified sonographer can help eliminate this bias. In addition, a meticulous and objective evaluation of the sonographer's proficiency and methodology can contribute substantially to resolving whether the limitations of ultrasound stem from operator skill or technical constraints.

Moreover, this study primarily focuses on preoperative imaging and immediate surgical outcomes. Longer-term follow-up data related to postoperative complications, recurrence rates, and patient quality of life would provide a more comprehensive assessment of the effectiveness of imaging strategies.

## Conclusions

In conclusion, a dual-imaging strategy involving 99mTC-Sestamibi SPECT/CT and US should be retained as the primary approach for adenoma localization, provided there are no contraindications. This recommendation is underpinned by the strategy's superior sensitivity rates and its inherent absence of additional patient or surgical-related risks. The PPV of adenoma detection in patients with disease localized on SPECT/CT and US is comparable to patients with disease localized on SPECT/CT only. Additionally, when juxtaposed with the dual imaging cohort, the SPECT/CT uni-localized group exhibits higher specificity rates, likelihood ratios for adenoma detection, and an overall cure rate.

Currently, in our institution, the decision to proceed with MIP is contingent upon dual localization on both imaging modalities. This analysis challenges the prevailing practice in our institution suggesting that SPECT/CT alone is sufficient for directing MIP in the presence of a negative ultrasound. The scope of the article is constrained by its reliance on retrospective data from a single institution. Consequently, to more effectively underscore the efficacy of a single imaging strategy with SPECT/CT, it is imperative that further multicenter and prospective studies be performed.
